# Constraint-based modeling of yeast mitochondria reveals the dynamics of protein import and iron-sulfur cluster biogenesis

**DOI:** 10.1016/j.isci.2021.103294

**Published:** 2021-10-15

**Authors:** Carl Malina, Francesca Di Bartolomeo, Eduard J. Kerkhoven, Jens Nielsen

**Affiliations:** 1 Department of Biology and Biological Engineering, Chalmers University of Technology, 412 96 Gothenburg, Sweden; 2 Wallenberg Center for Protein Research, Chalmers University of Technology, 41296 Gothenburg, Sweden; 3 Department of Biotechnology and Nanomedicine, SINTEF Industry, 7465 Trondheim, Norway; 4 Novo Nordisk Foundation Center for Biosustainability, Chalmers University of Technology, 412 96 Gothenburg, Sweden; 5 Novo Nordisk Foundation Center for Biosustainability, Technical University of Denmark, 2800, Kgs. Lyngby, Denmark; 6 BioInnovation Institute, Ole Måløes Vej 3, 2200 Copenhagen N, Denmark

**Keywords:** Cellular physiology, Cell biology, Integrative aspects of cell biology, Systems biology, In silico biology

## Abstract

Mitochondria are a hallmark of eukaryal cells and play an important role in cellular metabolism. There is a vast amount of knowledge available on mitochondrial metabolism and essential mitochondrial functions, such as protein import and iron-sulfur cluster biosynthesis, including multiple studies on the mitochondrial proteome. Therefore, there is a need for *in silico* approaches to facilitate the analysis of these data. Here, we present a detailed model of mitochondrial metabolism S*accharomyces cerevisiae*, including protein import, iron-sulfur cluster biosynthesis, and a description of the coupling between charge translocation processes and ATP synthesis. Model analysis implied a dual dependence of absolute levels of proteins in protein import, iron-sulfur cluster biogenesis and cluster abundance on growth rate and respiratory activity. The model is instrumental in studying dynamics and perturbations in these processes and given the high conservation of mitochondrial metabolism in humans, it can provide insight into their role in human disease.

## Highlights

• Reconstruction of mitochondrial protein import and cofactor metabolism in yeast
• Quantification of the energy cost of metabolite transport
• Protein import activity depends on growth rate and respiratory activity
• Quantification iron-sulfur cluster requirements show growth rate dependence

## Graphical abstract

**Figure:**
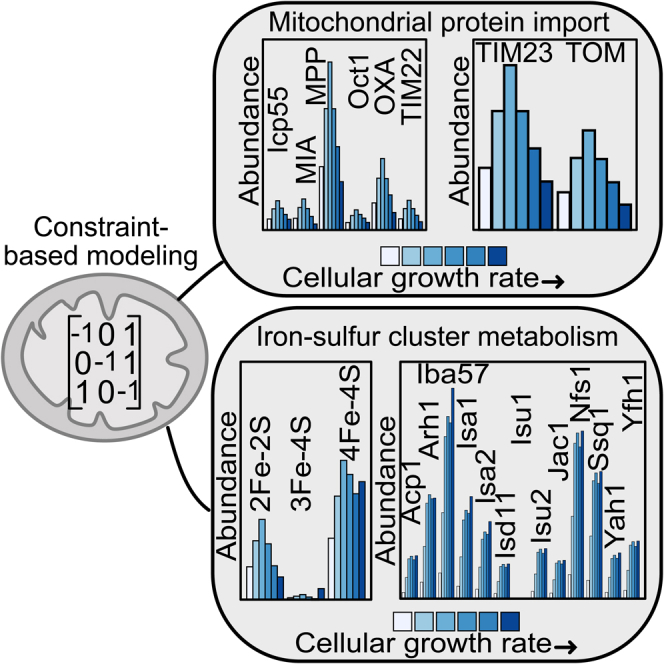

## Introduction

Mitochondria are a hallmark of eukaryal cells and essential for their viability. Being the center of oxidative phosphorylation, they are often referred to as the powerhouses of the cell, but mitochondria are also central to other parts of cellular metabolism and signaling (Malina et al., 2018). Mitochondria consist of two membranes, maintaining structural integrity and allowing generation of a proton motive force (PMF) that drives ATP synthesis. Only 1% of the mitochondrial proteins are encoded in the mitochondrial genome, whereas the remaining 99% are encoded by nuclear genes and rely on dedicated machinery for protein import (Wiedemann and Pfanner, 2017).

Mitochondrial proteomics studies have identified a large fraction of the mitochondrial proteome (Reinders et al., 2006; Sickmann et al., 2003; Vögtle et al., 2012; Vögtle et al., 2017; Zahedi et al., 2006), elucidating numerous mitochondrial functions beyond energy metabolism. These functions include protein import (Wiedemann and Pfanner, 2017) and synthesis of iron-sulfur (Fe/S) clusters (Lill and Freibert, 2020), both essential for cell viability under all conditions. Although essential, in the model eukaryote *Saccharomyces cerevisiae* protein import and Fe-S cluster biosynthesis make up only 3-5% and <1% of mitochondrial proteome mass (Di Bartolomeo et al., 2020; Morgenstern et al., 2017), whereas proteins engaged in these processes remain rather constant in absolute copy number as cells shift from fermentative to respiratory metabolism (Pfanner et al., 2019). The only moderately altered expression poses a challenge when it comes to experimentally determining the actual levels of proteins involved in Fe/S cluster generation and protein import required to sustain functional mitochondria.

Genome-scale models (GEMs) of metabolism are *in silico* representations of all metabolic reactions occurring in an organism (Nielsen, 2017). In these models, reactions are defined and linked to their respective catalyzing enzymes. GEMs can simulate phenotypes through flux balance analysis (Orth et al., 2010), where a cellular objective is used to achieve realistic flux distributions. Growth maximization is the most commonly used objective in microorganisms, represented by a biomass reaction that defines all macromolecular constituents of the cell (Feist and Palsson, 2010). Vital to the biomass reaction is the definition of the growth-associated energy cost (GAEC); partly consisting of the polymerization cost of the macromolecules; partly an amalgamation of other growth-related ATP costs that are more difficult to define (Thiele and Palsson, 2010).

Here, we present a detailed model representing various mitochondrial processes, including protein import, Fe/S cluster biosynthesis, and a detailed representation of the coupling between charge translocation and ATP synthesis. Combined this allowed us to gain new insight into the requirements, energetics and dynamics of mitochondrial protein import and Fe/S biosynthesis. The requirements of both mitochondrial protein import and Fe/S cluster biogenesis are dependent on the state of cellular metabolism, including the respiratory activity and the growth rate. This model is instrumental to study perturbations of these essential mitochondrial processes, and given the high degree of conservation in humans, this can provide insights into the function of these systems and their coupling to human disease.

## Results and discussion

### Increasing the scope of the yeast mitochondrial metabolic network

**Figure 1:**
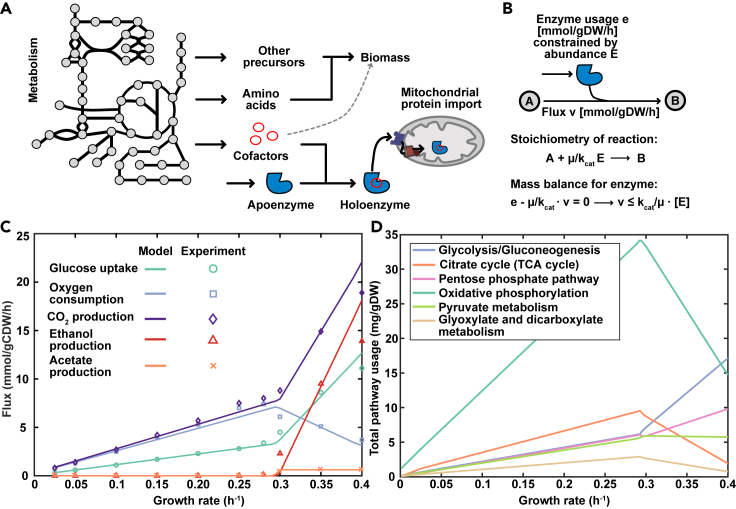
Overview of model and initial validation of model performance (A) Scope of the model. In addition to metabolism, the model accounts for the synthesis and binding of cofactors, including heme, iron-sulfur (Fe/S) clusters, lipoic acid and biotin, as well as mitochondrial protein import. The dotted line signifies the inclusion of cofactor requirements of unmodeled proteins into the biomass reaction. (B) Coupling constraint. The mathematical relationship between metabolic rates and enzyme usage is established based on a steady-state assumption. *k*_cat_, turnover rate; [E], enzyme abundance; μ, growth rate; v, reaction flux. (C) Model predicted exchange fluxes at increasing growth rates compared to experimental data from (van Hoek et al., 1998). (D) Pathway usage for the main pathways of central metabolism at increasing growth rates.

To increase the scope and predictive power of the mitochondrial metabolic network of *S*. *cerevisiae*, a GEM with an accurate representation of mitochondrial protein import and Fe/S cluster biosynthesis was created based on biochemical data, leveraging the consensus GEM Yeast8 (Lu et al., 2019), as outlined in the following sections. This culminated in the ecMitoYeast model (Figure 1A), which was generated using the GECKO framework (Sánchez et al., 2017) that incorporates enzymatic capacities by constraining metabolic reactions with the levels of their corresponding enzyme multiplied by its turnover number (*k*_cat_), as such limiting the flux through each reaction to physiologically feasible values (Figure 1B). In addition to the newly added processes, we also included reactions for incorporating biotin and the mitochondrially synthesized cofactors lipoic acid, Fe/S clusters, and the different forms of heme, into enzymes (Figure 1A).

**Figure 2:**
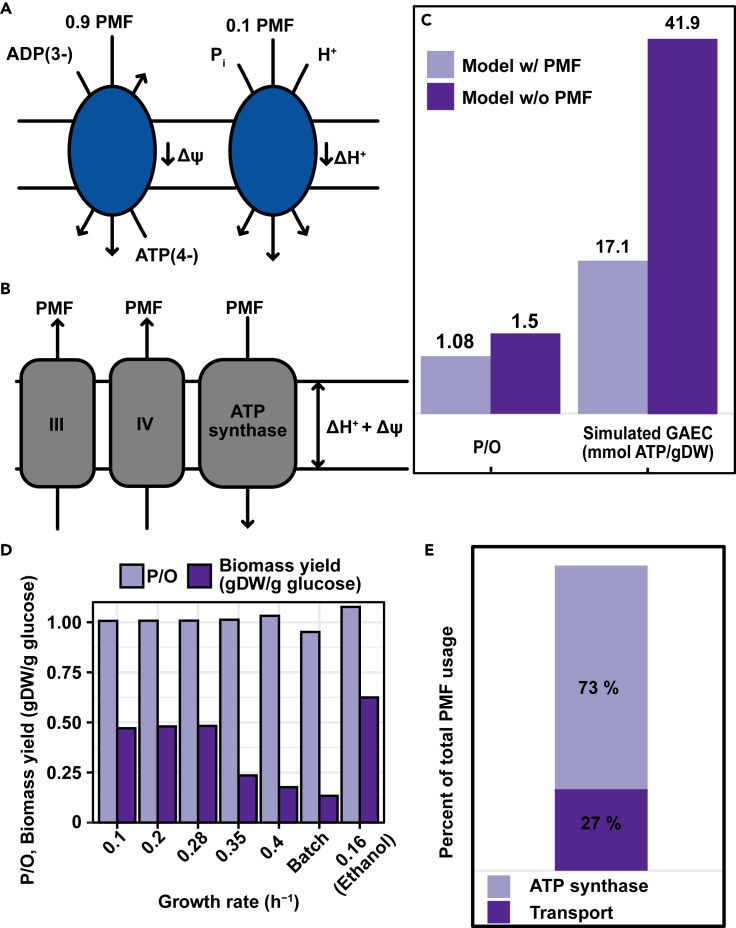
Model quantification of the proton motive force (PMF) (A) Coupling of reactions transporting charges or protons across the inner mitochondrial membrane to the PMF as exemplified by the ADP/ATP translocase, resulting in net transfer of a negative charge, and electroneutral but proton coupled phosphate carrier. (B) Coupling of the respiratory chain complexes and ATP synthase to the proton motive force. The components transfer PMF corresponding to the number of protons translocated across the inner membrane. (C) Effect of explicitly modeling the PMF on the operational phosphate/oxygen ratio (P/O) and the simulated growth-associated maintenance cost (GAEC). (D) Overall fraction of the generated PMF used for transport across the inner membrane, and synthesis of ATP. (E) P/O ratio and biomass yield at increasing growth rate. Ethanol indicates that simulations were carried out with ethanol as a carbon source.

To validate the overall performance of the model, we compared the major exchange fluxes at increasing growth rate to experimental data (Van Hoek et al., 1998). Growth is defined through the biomass reaction, which represents the metabolic requirements for the synthesis of biomass (Thiele and Palsson, 2010). The biomass constituent fractions are represented in mmol/gram dry weight (g DW) of cells and sums up the mol fractions required to produce 1 g DW, resulting in the biomass reaction having the unit h^−1^, which represents the specific growth rate. The model correctly predicted the metabolic shift to aerobic fermentation, coupled to a rearrangement of protein allocation from oxidative phosphorylation to glycolysis (Figures 1C and 1D) and a decrease in biomass yield (g DW/glucose) (Figure 2E). Interestingly, the model predicts a similar respiratory activity at dilution rates 0.1 h^−1^ and 0.4 h^−1^ (Figure 1C) indicating that at growth rates in chemostats, cells rely on both glycolysis and oxidative phosphorylation for ATP production. This mixed respiro-fermentative metabolism at high growth rates has been observed in *S*. *cerevisiae* (Cortassa and Aon, 1998; van Hoek et al., 2000).

### Representing the proton motive force

The proton motive force (PMF), essential in powering mitochondrial ATP generation, is primarily achieved by the complexes of the respiratory chain translocating protons across the mitochondrial inner membrane. This is driven by energy released from the passing of electrons from NADH and FADH_2_ to, ultimately, oxygen. In yeast, respiratory chain complex I is absent, and re-oxidation of NADH is carried out by two separate internal and external NADH dehydrogenases. These dehydrogenases lack the ability of proton translocation, and the translocation of protons therefore occur mainly at complexes III and IV of the respiratory chain. However, the PMF is also affected by other proton translocating processes, in addition to the transport of charged metabolites across the mitochondrial membrane. This has previously not been considered in models of yeast metabolism and to elucidate the contribution of the different processes, we set out to investigate the energetics of the PMF. The PMF consists of two components: a proton gradient (ΔH^+^) and a membrane potential (Δѱ) (Mitchell, 1961). In the model we introduced a PMF pseudo-metabolite that is co-transported in all steps that transport charged metabolites or protons across the mitochondrial membrane, inspired by a model of human central metabolism (Smith et al., 2017). This pseudo-metabolite serves to introduce a separation between the protons involved in chemical reactions within a cellular compartment from those that are crossing between compartments. From a modeling point of view, this is necessary to couple the proton translocating processes, including the respiratory chain complexes and transporters, to ATP synthesis. This coupling is an essential feature of energy metabolism (Mitchell, 1961), and is therefore of importance for inclusion in genome-scale metabolic models. In yeast, experimental measurements on isolated mitochondria, using NADH and ethanol as substrates, have shown the average relative contribution of Δѱ and ΔH^+^ to the total PMF is 90% and 10%, respectively (Beauvoit et al., 1989). Therefore, the contribution of reactions translocating charged metabolites and or protons, thus affecting either of the components was considered as such in the model with regards to the PMF pseudo-metabolite (Figures 2A and 2B). The coupling of proton translocating processes to ATP synthesis represents an advance in modeling energy metabolism in the format of a GEM. However, given the current scope of these models, the translocation of ions such as potassium and magnesium across the mitochondrial inner membrane, which has an important role in the PMF, is not modeled. Furthermore, a detailed description of the kinetics of mitochondrial energy metabolism in yeast is currently unavailable. It is therefore difficult to computationally estimate the PMF in yeast, albeit future developments in kinetic modeling of yeast might bring more insight into mitochondrial energy metabolism.

We analyzed the effect of explicitly coupling charge translocation to ATP synthesis on the phosphate/oxygen ratio (P/O), which is defined as the number of ATP synthesized per electron pair transferred through the electron transport chain to the final electron acceptor oxygen. We observed a decrease in the operational phosphate/oxygen ratio (P/O) from the theoretical maximum of 1.5 to 1.08 (Figure 2C), which is close to the experimentally measured *in vivo* P/O of about 1 (Verduyn et al., 1991). The P/O ratio was further found to remain constant at growth rate 0.1 h^−1^ to 0.4 h^−1^, while being lower at max growth rate, and higher at max growth rate with ethanol as carbon source (Figure 2D). The model also showed that metabolite transport across the mitochondrial inner membrane is a significant ATP cost, as the growth-associated energy cost (GAEC) was reduced roughly 60% (Figure 2C). In GEMs, the GAEC, also referred to as growth-associated maintenance, is a cost introduced to represent the energy required for processes related to growth. One part of this energy cost stems from the energy required for polymerization of the macromolecular building blocks of biomass, which can be calculated based on the biomass composition. The other part of the GAEC, referred to here as the simulated GAEC, is a lumped energy cost from growth-related processes other than polymerization of macromolecules, such as the maintenance of membrane potentials and turnover of macromolecules (Förster et al., 2003). The ATP requirement of these processes is difficult to measure experimentally and is therefore estimated by fitting the model to experimentally data. It is thus an artificial cost introduced to account for energy costs of processes not (yet) considered in the model. By expanding the scope of the yeast model to include the coupling between proton translocating processes and ATP synthesis, a larger fraction of the estimated GAEC can be accounted for (Figure 2C), which represents a significant improvement in the scope of model predictions. As the scope of the model increases, the reconstructed content will account for a larger fraction of the energy cost associated to growth, which therefore leads to a reduction in the simulated GAEC as seen in other studies expanding the scope of models (Liu et al., 2014; Oftadeh et al., 2021). Furthermore, transporting metabolites into mitochondria requires a large fraction of the generated PMF, which can be calculated using our model to be 27% at a growth rate of 0.1 h^−1^ (Figure 2E). This cost arises mainly from the fact that for each ATP produced, the exchange of mitochondrial ATP for cytosolic ADP leads to a net transport of a negative charge across the inner membrane, and that the import of phosphate to the mitochondrial matrix is proton-coupled. This leads to a reduction of the PMF and is thus associated with a cost on ATP production. For every ATP produced, the total cost would then add up an extra proton, corresponding to one PMF pseudo-metabolite in the model. This means that 4 protons, or 4 PMF pseudo-metabolites in our model, are required to synthesize one molecule of ATP, one for the import of ATP and phosphate, and 3 for driving ATP synthesis. This would explain the roughly 25% of the generated PMF pseudo-metabolites being used for transport.

### Reconstructing mitochondrial protein import

**Figure 3:**
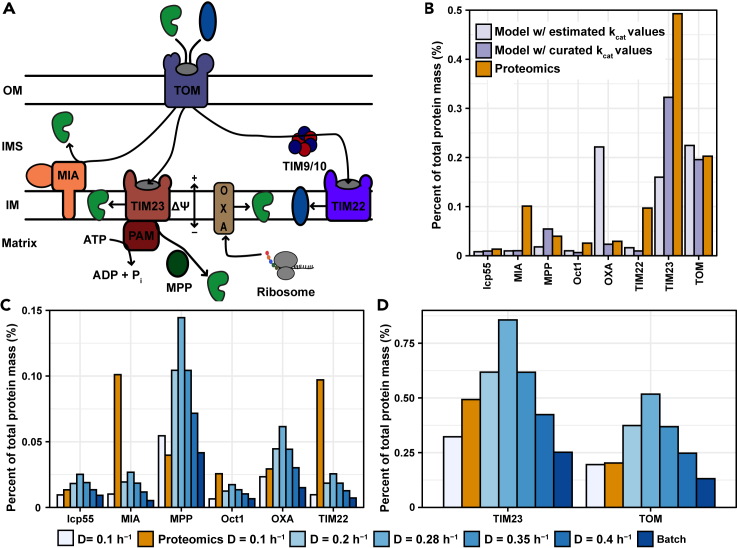
Modeling and quantification of mitochondrial protein import (A) Overview of the import pathways represented in the model. The translocase of the outer membrane (TOM) serves as the main point of entry for the vast majority of proteins. Import of ∼70% of mitochondrial proteins synthesized with cleavable positively charged N-terminal presequences, follows the presequence pathway mediated by the translocase of the inner membrane 23 (TIM23) complex. Import of the presequences, that are cleaved off by mitochondrial matrix processing protease (MPP) upon import (Yang et al., 1988), is driven by the membrane potential (Δѱ) (Truscott et al., 2001) and depending on the specific signals in the protein, the protein is either laterally inserted into the membrane in a Δѱ-dependent manner (van der Laan et al., 2007) or further imported into the matrix, driven by ATP hydrolysis by the presequence translocase-associated motor (PAM) (Horst et al., 1997). Intermembrane space proteins containing specific cysteine motifs forming disulfide bridges are imported by the mitochondrial intermembrane space assembly (MIA). Mitochondrial carriers are imported by the translocase of the inner membrane 22 (TIM22) complex, assisted by TIM chaperones. (B) Comparison of predicted abundance of the protein import machinery, before and after curation of *k*_cat_ values, to proteomics data at a growth rate of 0.1 h^−1^ (Lahtvee et al., 2017). (C and D) Predicted abundances of the protein import machinery at increasing growth rate and maximum growth rate. See also Figures S1 and S2

Mitochondrial import of proteins synthesized in the cytosol is essential for the viability of mitochondria and depending on the final location and properties of the protein, this involves one of five pathways (reviewed in Wiedemann and Pfanner, 2017). While a wealth of literature exists on protein import, much of which in yeast, to date this has not been comprehensively examined in a computational model. Leveraging advances in enzyme-constrained modeling, where enzymes are represented in metabolic reactions, we reconstructed protein import pathways. By focusing on metabolism, three main pathways were included (Figure 3A): (i) the disulfide relay through the mitochondrial intermembrane space assembly (MIA) (Bihlmaier et al., 2007), (ii) the translocase of the inner membrane 22 (TIM22) mediated pathway for import of mitochondrial carriers (Sirrenberg et al., 1996), and (iii) the translocase of the inner membrane 23 (TIM23) mediated presequence pathway which imports ca. 60-70% of the mitochondrial proteins (Schulz et al., 2015; Vögtle et al., 2009).

To represent protein translocation, all proteins in the model were assigned to a compartment based on experimental localization evidence. The compartmentalized enzymes were then assigned to a translocation pathway based on experimental data, protein properties and compartment, and the ATP cost of matrix import was defined from literature data. The proteins involved in protein import were added to the model, using *k*_cat_ values estimated from proteomics data (Ho et al., 2018) and a curated list of mitochondrial proteins (Table S1).

As initial model validation, we compared the predicted levels of protein import components to proteomics data at a growth rate of 0.1 h^−1^ (Lahtvee et al., 2017) (Figure 3B; Figures S1 and S2), yielding a significant correlation between model predictions and proteomics data (Pearson's R = 0.73, p value = 0.039, Figure S2A), upon curation of the initial *k*_cat_ values, with the TIM22 complex and the MIA as notable outliers with lower *in silico* levels. Excluding TIM22 and the disulfide relay, we obtained a good correlation between *in silico* and experimental data (Pearson's R = 0.94, p value = 0.0059, Figure S2B). TIM22 is involved in import of mitochondrial carriers and translocases, which are typically not included in metabolic models, much because of the scarcity of kinetic data on mitochondrial carrier proteins. Therefore, we initially only included the import of TIM23 subunits, for which *k*_cat_ values were collected from the literature. In a recent study, in which a model of metabolism and gene expression in yeast was reconstructed (Oftadeh et al., 2021), carrier proteins were incorporated in the model using the average *k*_cat_ of metabolic enzymes, corresponding to 70.9 s^−1^ (Sánchez et al., 2017). Using the same turnover number, we added the metabolite carriers to ecMitoYeast and studied the effect on the predicted protein levels of import components. Accounting for the metabolite carriers, the model performance for predicting the abundance of the TIM22 complex improved significantly, from predicting a 10-fold lower abundance to approximately a 2-fold difference between model predictions and experimentally measured values (Figure S4). We further observed a slight increase in the abundance of TOM, resulting from the requirement of the complex in import of the TIM22 subunits. However, we observed no other significant changes in the overall results of the model simulations. The low *in silico* levels of the MIA could be explained by many of its protein substrates being involved in copper homeostasis, respiratory chain complex assembly and maintenance of mitochondrial structure and function (Cavallaro, 2010; Longen et al., 2009; Vögtle et al., 2012), processes which are not explicitly represented in the model. Furthermore, the total ATP cost of protein import at a growth rate of 0.1 h^−1^ could be calculated as 4.7∗10^−3^ mmol ATP/gDW for importing 4∗10^−4^ mmol/gDW of mitochondrial matrix proteins. Although relatively small compared to the total energy demand of the cell, the average cost is 15 ATP per protein imported to the matrix.

Next, we analyzed the levels of the import components at an increasing growth rate (0.1–0.4 h^−1^) and including maximum growth (Figures 3C and 3D). Levels of the import components increase up to the growth rate just before onset of fermentation (0.28 h^−1^), from allocating 0.63% (0.1 h^−1^) to 1.67% (0.28 h^−1^) of the total cellular protein mass, whereas the proteomics data indicated an allocation of 1% (0.1 h^−1^). After the onset of fermentation (>0.28 h^−1^), the levels of import components decrease, in line with cells shifting toward fermentative metabolism (Figures 3C and 3D). Interestingly, the predicted levels of protein import components at 0.4 h^−1^ were higher than at 0.1 h^−1^, indicating that the respiratory activity required to sustain respiro-fermentative growth at high growth rate is even higher than at fully respiratory growth at low growth rate (cf. Figures 1C, 3C and 3D). Given the experimental observations that the levels of protein import required is only slightly altered between fermentation and respiration, these results point toward regulation of the activity of protein import as has been shown for the regulation of TOM by cytosolic kinases (Schmidt et al., 2011), which would allow mitochondria to sustain the level of import required given a largely constant fraction of its proteome. At maximum growth rate, lower predicted levels were observed (∼0.47% of total cellular protein), in line with the higher degree of fermentative metabolism. Protein import has been shown to occupy ca. 5% of the mitochondrial proteome in fermentative growth and between 3 and 5% of the mitochondrial proteome in respiratory growth (Di Bartolomeo et al., 2020; Morgenstern et al., 2017), and the mitochondrial proteome occupies 20-30% and ∼10% of total cell protein mass in respiration and fermentation, respectively (Di Bartolomeo et al., 2020; Pfanner et al., 2019). Based on these data, protein import would occupy ∼0.5% of the cellular proteome in fermentative conditions and ∼1.5% during respiration, which is in good agreement with the model predictions. The model predictions support the findings of a constant allocation of protein import. However, there is a discrepancy in the allocation of protein import in respiratory conditions comparing our previous study (Di Bartolomeo et al., 2020) to the study by Morgenstern et al. We found a constant fraction of the mitochondrial proteome allocated protein import when comparing fermentative conditions with glucose as carbon source to respiratory conditions during the ethanol phase of batch cultivation. On the other hand, Morgenstern et al. observed only a modest increase in the copy numbers of proteins in protein import in respiratory conditions, with glycerol as carbon source, compared to fermentative conditions, and consequently a decrease in mitochondrial proteome allocation of the protein import machinery. It is possible that part of this difference is explained by the different carbon sources used in the studies and the resulting differences in mitochondrial metabolism. The modest increase in copy numbers of protein import complexes as the mitochondrial proteome doubles in size would support the existence of post-translational modifications increasing the activity allowing the same copy numbers to support an increasing rate of import, as shown experimentally for the TOM complex (Schmidt et al., 2011). It would be interesting to evaluate the model predictions considering proteomics data from additional conditions, including different growth rates and carbon sources, as such data becomes available to further elucidate the dynamics of the mitochondrial proteome.

### Representing cofactor biosynthesis and incorporation in enzymes

Mitochondria are involved in the metabolism of a variety of cofactors that are important in a number of cellular processes. Among these cofactors are lipoic acid, biotin, the different forms of heme, and iron-sulfur clusters (addressed below). Firstly, lipoic acid is a sulfur-containing cofactor that is required for the function of pyruvate- and α-ketoglutarate dehydrogenase, and the glycine cleavage system (Schonauer et al., 2009). Second, biotin is an essential cofactor for the cytosolic (Acc1) and mitochondrial (Hfa1) acetyl-CoA carboxylases, pyruvate carboxylases (Pyc1 and Pyc2), and urea carboxylase (Dur1,2). Lastly, the different forms of heme, which are partly synthesized in mitochondria, are required for mitochondrial respiration and various cellular biosynthetic functions. In order to accurately represent mitochondrial metabolism, it is therefore important to detail the synthesis of these cofactors and their incorporation in target enzymes. For this, we generated a list of cofactor-containing enzymes (Table S3) based on literature. For the proteins considered in the model, a reaction incorporating the cofactor into the respective apo-protein was included (Figure 1A), whereas the cofactor requirement for the remaining unmodeled proteins was appended to the biomass reaction relative to the abundances of the individual proteins from proteomics data.

**Figure 4:**
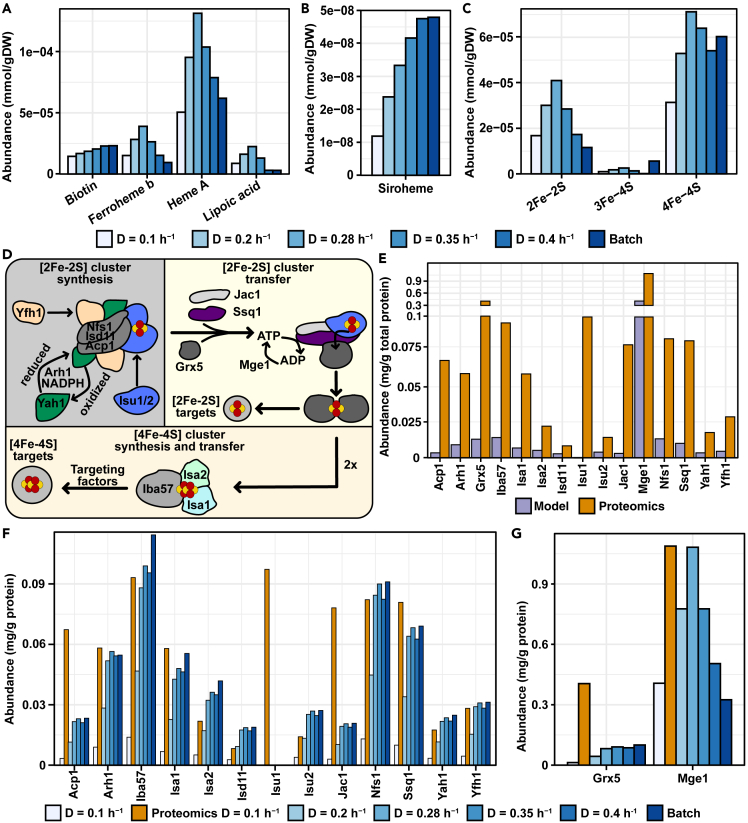
Modeling and quantification of cofactor requirements and abundances of proteins in iron-sulfur (Fe/S) cluster biosynthesis Predicted requirement of (A) biotin, ferroheme B, heme A and lipoic acid, and (B) siroheme and (C) iron-sulfur clusters at increasing growth rate. (D) Overview of mitochondrial iron-sulfur (Fe/S) cluster biosynthesis in yeast. Synthesis can be divided into three steps, the first being the synthesis of a [2Fe-2S] cluster on a scaffold protein (Isu1 or Isu2) which is essential for synthesis of all clusters. The [2Fe-2S] cluster is then transferred to glutaredoxin 5 (Grx5), followed by the insertion of the cluster into target apo-proteins or further transfer to the machinery responsible for synthesizing [4Fe-4S] clusters. (E) Comparison of predicted abundances of the Fe/S cluster biosynthetic machinery and proteomics data at a growth rate of 0.1 h^−1^ (Lahtvee et al., 2017). (F-G) Predicted abundances of the mitochondrial Fe/S cluster biosynthetic machinery at increasing growth rates. See also Figure S3

The requirements of lipoic acid, biotin and heme at increasing growth rate (0.1–0.4 h^−1^) and maximum growth rate (Figures 4A and 4B). The requirements of lipoic acid and ferroheme b are scaled with respiration, in line with their role in proteins of the respiratory chain and tricarboxylic acid (TCA) cycle, whereas increased fermentation at higher growth rates reduced their requirement. Heme a requirement partially scaled with respiration, although the *in silico* levels were higher at 0.4 h^−1^ than at fully respiratory growth at 0.1 h^−1^. In contrast to ferroheme b, heme a is a cofactor in both respiratory and biosynthetic proteins, the abundance of which scale with the increased biosynthetic need at faster growth. On the other hand, requirements of biotin and siroheme only scaled with growth rate. Siroheme is only required by Met5, involved in biosynthesis of methionine, and showed a low requirement (Figure 4B). The correlation of biotin requirements with growth rate can be explained by biotin containing proteins being involved in biosynthetic processes (Acc1 and Hfa1) and in intermediary metabolism (Pyc1 and Pyc2).

### Reconstructing Fe/S cluster biosynthesis

Iron-sulfur (Fe/S) clusters are versatile cofactors that are required for the viability of essentially all living organisms, including through their role in mitochondrial metabolism. A detailed description of the Fe/S cluster biosynthetic pathway is therefore imperative for a model of mitochondria. Mitochondrial Fe/S biosynthesis can be divided into three main steps (Figure 4D) (as reviewed in (Lill and Freibert, 2020)), and these were added to the ecMitoYeast model as described above. A list of Fe/S cluster containing proteins allowed prediction of cellular Fe/S requirements (Table S3). For those proteins that were considered in the model, a reaction incorporating the Fe/S cluster into the respective apo-protein was included (Figure 1A), whereas for the remaining unmodeled proteins their Fe/S cluster requirements were again appended to the biomass reaction.

The levels of proteins involved in Fe/S cluster biosynthesis are only moderately altered in their overall copy number in respiratory and fermentative conditions, indicating that the cell keeps a preparedness for conditions where the requirement of Fe/S clusters is high (Di Bartolomeo et al., 2020; Morgenstern et al., 2017; Pfanner et al., 2019). We used the model to predict the abundances of proteins involved in Fe/S cluster biosynthesis and compared those against abundances from proteomics data (Lahtvee et al., 2017). After curating the model to include *k*_cat_ values measured in physiologically relevant conditions (Supplementary Table S1), the predicted values were still lower (Figure 4E). The lower predicted requirement led us to further investigate the requirements and dynamics of Fe/S clusters and the proteins responsible for synthesizing them.

As above, we predicted the Fe/S cluster requirements at increasing growth rate (0.1–0.4 h^−1^) and including maximum growth (Figure 4C). The predicted requirements for [2Fe-2S] clusters scaled well with respiration (cf. Figures 4C and 1D), as most proteins containing [2Fe-2S] clusters are involved in mitochondrial energy metabolism and increasing fermentation at higher growth rates reduced [2Fe-2S] cluster requirements. Contrastingly, [4Fe-4S] clusters showed a less drastic decrease in their requirement at higher growth rates, including maximum growth when fermentation is predominant. With the exception of Sdh2, proteins containing [4Fe-4S] clusters are involved in biosynthetic processes that are up-regulated at higher growth rates (Metzl-Raz et al., 2017; Voichek et al., 2016). Corroborating this, the levels of individual Fe/S containing proteins indicated a higher abundance of amino acid biosynthetic proteins Ilv3, Leu1 and Lys4, and a lower demand of respiratory chain proteins Rip1 and Sdh2 in the model (Figure S3). Overall, the model predicted a gradual change in the composition of the pool of Fe/S clusters, from a relatively larger importance of [2Fe-2S] proteins involved in respiration, to a higher importance of [4Fe-4S] proteins involved in biosynthetic processes. This is in line with the previously observed balancing of the dual role of mitochondria in biosynthetic processes and energy generation as cells transition between fermentation and respiration (Di Bartolomeo et al., 2020).

Predicted levels of Fe/S cluster biosynthetic proteins increased until maximum respiration was reached (0.28 h^−1^), whereas even higher growth rates yielded only minor additional changes in protein levels (Figures 4F and 4G). The total requirement of Fe/S clusters synthesis is relatively constant at higher growth rates, and higher than at fully respiratory conditions at lower growth rates, as from 0.28 h^−1^ and higher the *in silico* levels were similar to those measured at a growth rate of 0.1 h^−1^. Meanwhile, a few proteins (i.e. Mge1, Grx5, Acp1, Jac1 and Isu1/2) showed different profiles than the overall Fe/S biosynthetic machinery. The highest level of Mge1 was predicted when the respiratory capacity was at its maximum (0.28 h^−1^), and subsequently decreased at higher growth rates. Mge1 is a nucleotide exchange factor that is shared between Fe/S biosynthesis and protein import, and the decreased requirements of the latter process at higher growth rates invoked the decreasing Mge1 requirement. The low predicted levels of Grx5 and Acp1 are likely because of their involvement in other processes that are not in the scope of our model. Grx5 has a role in response to oxidative-stress and osmotic stress (Rodríguez-Manzaneque et al., 1999), whereas Acp1 is involved in synthesis of mitochondrial fatty acids (mtFAS) and functions as an acetyl-CoA-dependent allosteric regulator of respiratory chain complex assembly (Van Vranken et al., 2018). The requirement of Jac1 was also seemingly low. The protein is involved in binding to Isu1/2 scaffold and stimulating the ATPase activity mtHsp70 protein Ssq1 in Fe/S cluster transfer. The fact that the model predictions in terms of Ssq1 were closer to proteomics data could indicate that the stoichiometry between Jac1 and Ssq1 is not 1:1. Another potential reason is that binding of Jac1 to Isu1/2 occurs at a lower rate than the ATP hydrolysis by Ssq1, the rate used for the transfer reaction, which would cause an underprediction of the Jac1 levels. Lastly, in ecMitoYeast the two isoforms of the Fe/S scaffold protein (Isu1/2) are interchangeable, leading to only one of them being used, thereby representing the total abundance. Interestingly, the levels of Fe/S biosynthetic proteins predicted at higher growth rates were more similar to the levels measured experimentally at 0.1 h^−1^ (Lahtvee et al., 2017), where the predicted cluster requirements were lower.

Taken together, this could indicate that cells maintain a spare capacity in Fe/S biosynthetic protein irrespective of requirements, in line with the essentiality of Fe/S cluster biosynthesis at all conditions. Alternatively, a low saturation of the enzymes involved in the synthesis of Fe/S clusters can explain the lower predicted levels. The model assumes a global enzyme saturation level, which could contribute to differences in the predicted and experimentally measured protein levels. The model relies on the enzyme turnover numbers as well as the mechanistic knowledge available. Although the biogenesis of Fe/S clusters has been given increasing attention in terms of elucidating the mechanism of synthesis, the data on enzyme turnover numbers in yeast are still scarce. Furthermore, many of the turnover numbers available are from *in vitro* studies and may not accurately represent the physiological conditions *in vivo*, as shown in studies on human Fe/S cluster biosynthesis (Gervason et al., 2019). The discrepancy between *in vitro* measured turnover numbers and *in vivo* activities of enzymes in yeast has been further shown in a recent study evaluating proteomics and flux data for 358 reactions under 26 conditions (Chen and Nielsen, 2021). Lastly, the list of Fe/S proteins accounted for in the model is based on the current list of known Fe/S proteins, which given the versatility of functions requiring Fe/S clusters is likely to be extended as the knowledge on cellular process of yeast is expanded. Improvements in model performance are therefore likely to come from biochemical studies on the mechanism of Fe/S cluster biogenesis to characterize the kinetics of the enzymes involved as well as to identify novel Fe/S clusters requiring proteins.

### The model can serve as a tool for studying mitochondrial metabolism

ere, we present ecMitoYeast, a detailed model of mitochondrial metabolism in the eukaryal model organism *S*. *cerevisiae*, including the essential processes of protein import and iron-sulfur (Fe/S) cluster biosynthesis, as well as a detailed representation of the coupling of charge translocation across the mitochondrial inner membrane to ATP synthesis. The model showed that the requirements of both mitochondrial protein import and Fe/S cluster biogenesis are dependent on the growth rate and the respiratory activity. Our findings support the experimental observation that a constant fraction of the mitochondrial proteome is allocated to protein import. Furthermore, our results indicate that cells keep a spare capacity in the machinery responsible for Fe/S cluster biosynthesis and the predictions on the cofactor requirement highlights the dual role of iron-containing cofactors in respiratory functions and biosynthesis. Our work provides insight into the dynamics of mitochondrial metabolism. The model developed could serve as a valuable tool to investigate mitochondrial function and, given the high conservation of mitochondrial metabolism in eukaryal cells, the role of mitochondrial dysfunction in human health.

Improvements of the predictive power of yeast genome-scale models will likely come from the development of a model of metabolism and expression (ME), where all the processes from expression of a gene to synthesis of its corresponding protein are represented along with an explicit modeling of transporters. The content reconstructed in this study can be used as a scaffold for protein import and cofactor metabolism in such a model. Further improvements will also come from additional experimental studies of these processes to elucidate the mechanistic details and turnover rates. In combination, this would allow a more accurate representation of mitochondrial metabolism.

### Limitations of the study

In the present model, we do not directly account for the processes of gene expression and translation. As both the cytoplasmic and mitochondrial transcriptional and translational machineries involve a large number of proteins, inclusion of these processes would significantly increase the scope of the model. It would be interesting to see what effects that would have on model performance. Furthermore, in the present model, assumptions about the rate of translocation for some of the import complexes were made due to lack of experimental data. Model predictions would likely benefit from incorporation of experimental data on the mechanism and catalytic rate of protein import complexes. Lastly, we validated the model predictions against experimental data at a dilution rate of 0.1 h^−1^ (Lahtvee et al., 2017), drawing advantage of the availability of quantitative data on both protein and transcript level, allowing the estimation of proteins not captured in the proteome dataset using transcriptome data. It would be interesting to perform a further validation against data from different growth rates, as such datasets become available.

## STAR★Methods

### Key resources table

**Table:** 

REAGENT or RESOURCE	SOURCE	IDENTIFIER
**Deposited data**		

Metabolic flux data at increasing growth rate	van Hoek et al. (1998)	Table 1 main text
Absolute mRNA and protein abundances, mRNA-protein ratio, lipid composition of biomass	Lahtvee et al. (2017)	Tables S2, S3, S4 and S12
Data on lipid composition of biomass	Ejsing et al. (2009)	Supplementary dataset D1
Absolute protein levels	Ho et al. (2018)	Table S4

**Software and algorithms**		

Code associated to the model construction and simulation. Code for analyzing results and generating figures.	This paper	https://github.com/SysBioChalmers/mitoYeast-GEM
MATLAB R2018b	The MathWorks Inc.	RRID:SCR_001622; https://www.mathworks.com/
COBRA toolbox v3.0	Heirendt et al. (2019)	https://github.com/opencobra/cobratoolbox
RAVEN toolbox 2.0	Wang et al., 2018	https://github.com/SysBioChalmers/RAVEN
GECKO toolbox	Sánchez et al. (2017)	https://github.com/SysBioChalmers/GECKO/
Rstudio v1.2.5042	Rstudio	RRID:SCR_000432; https://rstudio.com/products/rstudio/download/
ggplot2	Wickham (2016)	RRID:SCR_014601; https://ggplot2.tidyverse.org/
reshape2	GitHub	https://github.com/hadley/reshape
Ggpubr	CRAN	RRID:SCR_021139; https://cran.r-project.org/web/packages/ggpubr/index.html
RColorBrewer	CRAN	RRID:SCR_016697; https://cran.r-project.org/web/packages/RColorBrewer/index.html
IBM cplex v12.8.0	IBM	https://www.ibm.com/analytics/cplex-optimizer
Gurobi Optimizer 8.0.1	Gurobi Optimization, LLC.	https://www.gurobi.com/products/gurobi-optimizer/

### Resource availability

#### Lead contact

Further information and requests for resources should be directed to and will be fulfilled by the Lead Contact, Jens Nielsen (nielsenj@chalmers.se).

#### Materials availability

This study did not generate any new reagents.

### Method details

#### Construction of the model

##### Compartmentalization

The yeast consensus GEM (yeastGEM, https://github.com/SysBioChalmers/yeast-GEM; version 8.3.5) was used as a template upon which the model was built. As a first step to allow the reconstruction of mitochondrial protein import, two new compartments (the outer membrane and intermembrane space) were added to the model. A comprehensive list of mitochondrial proteins was generated based on localization evidence for mitochondrial localization of all genes in the model was evaluated using data from the MitoMiner database (Smith and Robinson, 2016), which combines GFP-tagging studies, mass-spectrometry mitochondrial proteomics and mitochondrial targeting sequence predictions with annotations from Gene Ontology. This localization evidence was compared to evidence from UniProt (The UniProt Consortium, 2019) and Saccharomyces genome database (Cherry et al., 2012) to generate a comprehensive list of mitochondrial proteins (Table S2). Next, we used the evidence to assign all mitochondrial proteins to one of the four mitochondrial compartments: outer membrane, intermembrane space, inner membrane and matrix. Proteins with a transmembrane component were assigned to the corresponding membrane. Complexes composed of multiple subunits were assigned to the compartment of its components, but if any of the subunits were localized to a membrane the entire complex was assigned to the membrane, in order to allow interactions with metabolites on both sides of the membrane. The compartment of reactions in the model was updated according to their localization of the corresponding enzymes.

##### Updating the lipid composition of biomass

The lipid composition of biomass was updated in order to achieve a more comprehensive list of the lipid species constituents of biomass. This was done by combining the datasets from two studies measuring the lipid composition (Ejsing et al., 2009; Lahtvee et al., 2017). Abundance values from the Ejsing et al. dataset, for lipid species not measured in the Lahtvee et al. dataset, were converted from mol/mol to mg/gDW using the 8 % lipid content measured in the Lahtvee and considering the fraction already measured in that dataset. The reference conditions of both studies were used.

##### Representing the proton motive force (PMF)

The PMF was represented in the model by introducing a pseudometabolite that is co-transported in all reactions transporting charged metabolites or protons across the inner membrane. The PMF consists of two components, a membrane potential (Δѱ) and a proton gradient (ΔH^+^). The relative contribution of Δѱ and ΔH^+^ were accounted for by co-transporting 0.9 PMF metabolites for transport steps affecting Δѱ and 0.1 PMF metabolites for steps affecting ΔH^+^. These values are the average of the experimentally determined relative contributions for *S*. *cerevisiae* (Beauvoit et al., 1989). We implemented the co-transport of PMF in all reactions of the model transporting charges across the membrane. The reactions representing respiratory chain complexes III and IV were set to transport PMF metabolites corresponding to the number of protons translocated from the matrix to the intermembrane space, as transporting protons affect both Δѱ and ΔH^+^. ATP synthase was set to transport 3 PMF metabolites per molecule of ATP produced, since 3 protons are required.

Since explicitly modeling the PMF results in an energy cost for transport of charged metabolites, such as ATP and phosphate across the inner mitochondrial membrane, the growth-associated energy cost (GAEC) was updated. Part of this cost represents an amalgamation of energy costs not related to polymerization of macromolecules, of which the transport of metabolites across membranes is included. To account for the energy cost associated with metabolite transport across the inner mitochondrial membrane, the non-polymerization related part of the GAEC was reformulated by fitting to experimental data. Furthermore, to enable adjustment of the P/O ratio, a PMF sink reaction, allowing the free passage of PMF pseudometabolite from the intermembrane space to the matrix was added.

##### Reconstruction of cofactor biosynthesis and usage

Reactions involved in the pathways for biosynthesis of iron-sulfur (Fe/S) clusters and lipoic acid were constructed based on information in literature about the mechanistic details. Information on the proteins requiring the modeled cofactors lipoic acid, biotin, Fe/S and heme, was obtained from the literature and the UniProt database (The UniProt Consortium, 2019) and was used to construct reactions incorporating the cofactor into the proteins modeled. For unmodeled proteins, an integrated proteomics data set of relative protein abundances from PaxDb (Wang et al., 2015), with a coverage of 96 % of yeast proteins was used to estimate the protein levels, and thereby the cofactor requirement. This requirement was then added to the biomass reaction.

Yeast contains a separate, less characterized, cytosolic machinery for Fe/S cluster synthesis, starting from a yet unknown precursor derived from mitochondrial Fe/S biosynthesis. Due to a lack of mechanistic details of cytosolic Fe/s synthesis, all clusters in the model were assigned to be synthesized in the mitochondrion. As the model initially predicted much lower protein levels of proteins involved in Fe/s biosynthesis than experimentally observed, we re-evaluated the *k*_cat_ values of the Fe/S cluster biosynthetic reactions. Many of the molecular mechanisms of Fe/S cluster biosynthesis have been characterized *in vitro*, often using the chemical reducing agent dithioreitol (DTT) instead of the electron transfer chain consisting of NADPH, adrenodoxin reductase homolog Arh1 and ferredoxin Yah1 that is used *in vivo* (Gervason et al., 2019). Therefore, we updated the model to only include *k*_cat_ values measured in physiologically relevant conditions (Gervason et al., 2019).

##### Reconstruction of protein import

Literature review and evaluating the scope of the model led to the identification of 3 main pathways for protein translocation to include in the model: (i) the disulfide relay for import of a set of proteins, containing specific cysteine motifs, into the intermembrane; (ii) the translocase of the inner membrane 22 (TIM22) pathway for import of mitochondrial carriers (Sirrenberg et al., 1996); and (iii) the translocase of the inner membrane 23 (TIM23)-mediated presequence pathway for import of proteins of the mitochondrial inner membrane and matrix containing a cleavable, positively charged N-terminal targeting sequence. Based on the sequence of events in these pathways, template reactions a set of mechanistic template reactions were constructed. In the presequence pathway, import of the presequence is driven by the membrane potential across the inner membrane, and further import into the matrix is driven by repeated cycles of ATP hydrolysis. Upon import, the presequence is cleaved off by the mitochondrial processing protease (MPP) (Yang et al., 1988). For import of the presequence-containing proteins of the inner mitochondrial membrane, two mechanisms of import exist (Bohnert et al., 2010); (i) the stop-transfer mechanism, in which proteins containing a hydrophobic segment in proximity of the N-terminal translocation is halted and the proteins are laterally inserted in the membrane (van der Laan et al., 2007) and; (ii) the conservative sorting mechanism, in which the entire proteins or specific transmembrane domains are first imported into the matrix and then inserted into the membrane by the OXA translocase (Stiller et al., 2016). Based on the list of mitochondrial localization and experimental evidence, proteins were assigned to a pathway. For inner membrane proteins, proteins with proline residues in the transmembrane segments were assigned to the conservative sorting pathway (Meier et al., 2005), while the remaining proteins were assigned to the stop transfer pathway. The sequence of the transmembrane domains was retrieved from UniProt (The UniProt Consortium, 2019) in the case where the sequence was available. Otherwise, the transmembrane domains of the inner membrane proteins were predicted using TMpred tool (ExPASy, Swiss Institute of Bioinformatics) using protein sequences obtained from UniProt.

The effect on the PMF of importing each presequence-containing protein of the mitochondrial inner membrane was calculated based on the amino acid sequence of the protein. The import reaction was then set to co-transport PMF metabolites corresponding to the total charge of the protein, where 0.9 PMF metabolites were transported per positive charge of the protein. The ATP cost for import of matrix proteins and proteins using the conservative sorting pathway was calculated based on the length of the protein or transmembrane sequence, respectively, and an average length between binding sites of mtHsp70 of 25 amino acids (Rüdiger et al., 1997) resulting in a cost of 1 ATP per 25 amino acids. Using this information and the template reactions defined for each pathway, reactions for translocating of each model protein into its correct compartment were added.

The initial set of *k*_cat_ values for each component of protein import was estimated using proteomics data (Ho et al., 2018) and a list of mitochondrial proteins and their corresponding pathway of import, according to the following equation:

(Equation 1) $$k_{cat}=\frac{\mu *\sum \left[E\right]}{\left[E\right]}\;$$

where Σ[E] is the summed abundance of all proteins requiring the import machinery component and [E] is the average abundance of the subunits of the import machinery component, under the assumption that the effect of protein degradation can be neglected.

Curation of *k*_cat_ values for the components of mitochondrial protein import was performed by querying the BRENDA enzyme database (Jeske et al., 2019) and literature for experimentally measured *k*_cat_ values for the import components. The translocation rate of the translocase of the outer membrane was assumed to operate at a rate equal to the translation rate, (10 s^-1^) (Arava et al., 2003), given the experimental observations that about a third of the mitochondrial proteins can be imported co-translationally (Becker et al., 2019). Furthermore, the OXA insertase was assumed to operate at the same rate since it is responsible for co-translational insertion of mitochondrially synthesized proteins. Furthermore, the translocation rate of the TIM23 complex, including the presequence associated import motor (PAM), was calculated based on an experimentally measured rate of 9 amino acids per second (Lim et al., 2001) and the median protein length in *S*. *cerevisiae* calculated from protein sequences obtained from UniProt (The UniProt Consortium, 2019).

##### Generation of ecMitoYeast

The ecMitoYeast model was generated based on the GECKO toolbox available at https://github.com/SysBioChalmers/GECKO. This framework relies on the RAVEN toolbox (Wang et al., 2018). Briefly, the algorithm used by GECKO, queries the BRENDA database (Jeske et al., 2019) to retrieve all the necessary *k*_cat_ for each reaction according to gene annotation, substrate and organism specificity. The *k*_cat_ values are then used to constrain the reactions according to:

(Equation 2) $$-\frac{1}{k_{cat}^{ij}}v_{j}+e_{i}=0$$

(Equation 3) $$0\leq e_{i}\leq \left[E_{i}\right]$$

(Equation 4) $$v_{j}\leq k_{cat}^{ij}\cdot \left[E_{i}\right]$$

where *v*_*j*_ represents the flux through reaction *j*, *e*_*i*_ represents the amount of enzyme *i* allocated to reaction *j*, *E*_*i*_ represents the concentration of enzyme *i* and *k*_*cat*_ represents the highest turnover number available for enzyme *i* and reaction *j*. The details of this procedure can be found in the supplementary material of the GECKO paper (Sánchez et al., 2017). As a result of the newly reconstructed content, the unit of enzyme usage was changed, resulting in updating Equations (Equation 2), (Equation 4) accordingly:

(Equation 5) $$-\frac{\mu }{k_{cat}^{ij}}+\;e_{i}=0$$

(Equation 6) $$v_{j}\leq \frac{k_{cat}^{ij}}{\mu }*\left[E_{i}\right]$$

#### Simulation details

Simulations were performed using the COBRA toolbox v3.0 (Heirendt et al., 2019) with solver IBM CPLEX v12.8.0. During construction of ecMitoYeast using the GECKO toolbox, solveLP from the RAVEN toolbox was used with solver Gurobi Optimizer v8.0.1.

##### Constraining the enzyme usage

The total amount of enzyme was limited by introducing a pseudometabolite that represents an aggregated pool of all enzymes in the model. The usage of this pseudometabolite is limited by the total protein content, P_tot_ (g/gDW), multiplied by the mass fraction, *f*, of enzymes accounted for by the model based on abundance data from PaxDB (Wang et al., 2015), and the average *in vivo* saturation of all enzymes, σ. In this study, P_tot_ of 0.46 g/gDW, an *f* of 0.446 g protein/g total cellular protein, and a σ, fitted to experimental data from aerobic chemostats, of 0.53 was used. For each enzyme in the model, a reaction that draws mass from the protein pool was introduced, resulting in the following mass balance for the enzyme pool:

(Equation 7) $$\overset{P}{\underset{i}{\sum }}MW_{i}e_{i}\leq \sigma \cdot f\cdot P_{tot}$$

Upon introducing the reconstruction of the additional mitochondrial processes, this mass-balance was updated to account for the growth rate:

(Equation 8) $$\overset{P}{\underset{i}{\sum }}\frac{MW_{i}}{\text{μ}}e_{i}\leq \sigma \cdot f\cdot P_{tot}$$

##### Chemostat growth simulations

For simulations of chemostat cultivations, the following procedure was used. Firstly, remove constraints on glucose uptake rate and set a minimal media. All simulations in this study were carried out with glucose as carbon source and ammonium as nitrogen source. Next, set the growth rate. Thereafter, to account for the usage of enzymes in reactions having the unit mmol/gDW/h and the protein pool being supplied to the model as g/gDW, multiply each coefficient of usage (Equation 2) by the growth rate and divide the coefficient of protein pool usage in the reaction drawing protein from the pool by the growth rate (Equation 7). Subsequently, the substrate uptake rate is minimized, fixed at the obtained values and the total enzyme usage was minimized, similarly to parsimonious FBA (Lewis et al., 2010). Lastly, some exchange reactions, including acetate and pyruvate were limited to experimental values, and the reversibility of some reactions involving NADPH were corrected based on a previous study (Pereira et al., 2016). The upper bounds of exchange fluxes were constrained according to experimental data (van Hoek et al., 1998).

##### Batch growth simulations

To predict the maximum growth rate with glucose as carbon source using the model, the following procedure was used. Firstly, remove constraints on glucose (or ethanol in the case where used as carbon source) and ammonium uptake. Next, set a minimal media allowing only the uptake of essential metabolites, such as sulfate and trace metals and ions. Thereafter, incorporate the growth rate in constraints, as described above. Lastly, perform a binary search minimizing the glucose uptake rate to find the maximum feasible growth rate, fix the optimal value of the glucose uptake rate and the maximum feasible growth rate and minimize the total protein usage. For simulations of maximum growth rate in batch conditions, that is maximum growth in excess glucose, the exchange rates of glycerol and acetate were constrained to experimentally determined levels (Heyland et al., 2009). For simulations of maximum growth rate with ethanol as a carbon source, the model was allowed free uptake of ethanol, followed by a maximization of the growth rate and a minimization of the total protein pool usage.

##### Simulations related to the P/O ratio and growth-associated energy cost (GAEC)

The P/O ratio represents the ATP yield of respiration and is a measure of the efficiency of the oxidative phosphorylation. The P/O ratio is calculated as the amount of ATP produced per pair of electrons entering the respiratory chain. The *in silico* P/O ratio was calculated in the following way. First, the glucose uptake rate was set to 1 mmol/gDW/h and hydrolysis of ATP was set as objective function. This objective function was maximized to simulate the maximum the production of ATP per molecule of glucose in the model. The P/O ratio was then calculated as the ratio between ATP produced by ATP synthase and the total number of electron pairs transferred to the respiratory chain at the two NADH dehydrogenase and succinate dehydrogenase.

The GAEC consists of the energy required for polymerization of macromolecules and the amalgamation other costs that are more difficult to define. The cost related to polymerization of macromolecules was calculated based on the biomass composition. The remaining part of the GAEC was estimated through fitting model predictions to experimental data from aerobic chemostats by minimizing the error in the prediction of exchange fluxes for glucose, oxygen carbon dioxide.

#### Data for model validation

The data used for validating the model predictions related to protein import and iron-sulfur cluster biosynthesis, was collected from a study performing absolute proteome and transcriptome analysis of *S*. *cerevisiae* CEN.PK113-7D grown in glucose-limited minimal media in aerobic chemostats a dilution rate of 0.1 h^-1^ (Lahtvee et al., 2017).

Many of the proteins involved in protein import are membrane bound. Membrane proteins are difficult to quantify due to their relative hydrophobicity and generally low solubility and low digestibility with trypsin. We therefore made use of both the proteomics data and the transcriptomics data for obtaining the protein abundances for the proteins involved in protein import. For proteins that were not detected in the proteomics dataset, we used the average mRNA-to-protein levels for the import complexes based on identified subunits to calculate the protein abundance from mRNA abundance.

Similar to for proteins involved in protein import, protein abundances iron-sulfur cluster biosynthetic proteins not detected in the proteome dataset were estimated using the mRNA levels and the mRNA-to-protein ratio.

### Quantification and statistical analysis

All statistical analysis were carried out in R. Any calculations related to the data used for validation of the model were carried out in Matlab or R.

## Data Availability

• The models and scripts generated using this study are available at GitHub (https://github.com/SysBioChalmers/mitoYeast-GEM). The model is available as a .mat file. In addition, users can contribute to further model development by posting issues or suggesting changes. • This study uses existing publicly available data for the validation of model performance. The sources of these data are listed in the key resource table.

## Supplemental information

Document S1. Figures S1–S4 and Table S1

Table S2. List of mitochondrial proteins used for construction of model, related to figure 1

Table S3. List of cofactor-containing proteins used for construction of model, related to figure 4
