# Supplementary material for: Constraint-based modeling of yeast mitochondria reveals the dynamics of protein import and iron-sulfur cluster biogenesis
Source: iScience. 2021 Oct 15;24(11):103294. doi: 10.1016/j.isci.2021.103294 (PMC8564123; doi:10.1016/j.isci.2021.103294)
Supplement: Document S1. — Figures S1–S4 and Table S1 [file mmc1.pdf]

iScience, Volume *24*

Supplemental information

Constraint-based modeling of yeast mitochondria reveals the dynamics of protein import and iron-sulfur cluster biogenesis

Carl Malina, Francesca Di Bartolomeo, Eduard J. Kerkhoven, and Jens Nielsen

Table S1. k_{cat} values used for protein import components, related to Figure 3B and STAR Methods. Estimated k_{cat} values denote values estimated based on proteomics data (Ho et al., 2018) and Curated k_{cat} denote values gathered from experimental studies.

Complex	Estimated k_{cat} (h^{-1})	Curated k_{cat} (h^{-1})	Note
**BCS1**	5.2034	100.5	Assumed to operate at translation rate (10 amino acids/second)
**MIA**	9.1313	9.1313	Estimated value used
**ICP55**	235.7786	212.4	Assume to operate at same rate as other processing peptidases (Oct1 and MPP)
**MIM**	11.3485	11.3485	Estimated value used
**MPP**	319.0199	212.4	Median value for EC3.4.24.64 from Human (PMID: 26082885)
**OCT1**	144.2625	212.4	Median value for EC3.4.24.59 from Human (PMID: 26082885)
**OXA**	11.8620	100.5	Assumed to operate at translation rate (10 amino acids/second)
**PAM**	95.0973	83.94	Lumped with TIM23. Calculated based on translocation rate of 9 amino acids/second (PMID: 11230118) and a median protein length of 358
**TIM chaperones**	5.7960	43.8168	Assumed to operate at same rate as TIM22. Estimated value used
**TIM22**	43.8168	43.8168	Estimated value used
**TIM23**	248.7712	83.94	Lumped with TIM23. Calculated based on translocation rate of 9 amino acids/second (PMID: 11230118) and a median protein length of 385
**TOM**	117.3856	100.5	Assumed to operate at translation rate (10 amino acids/second)

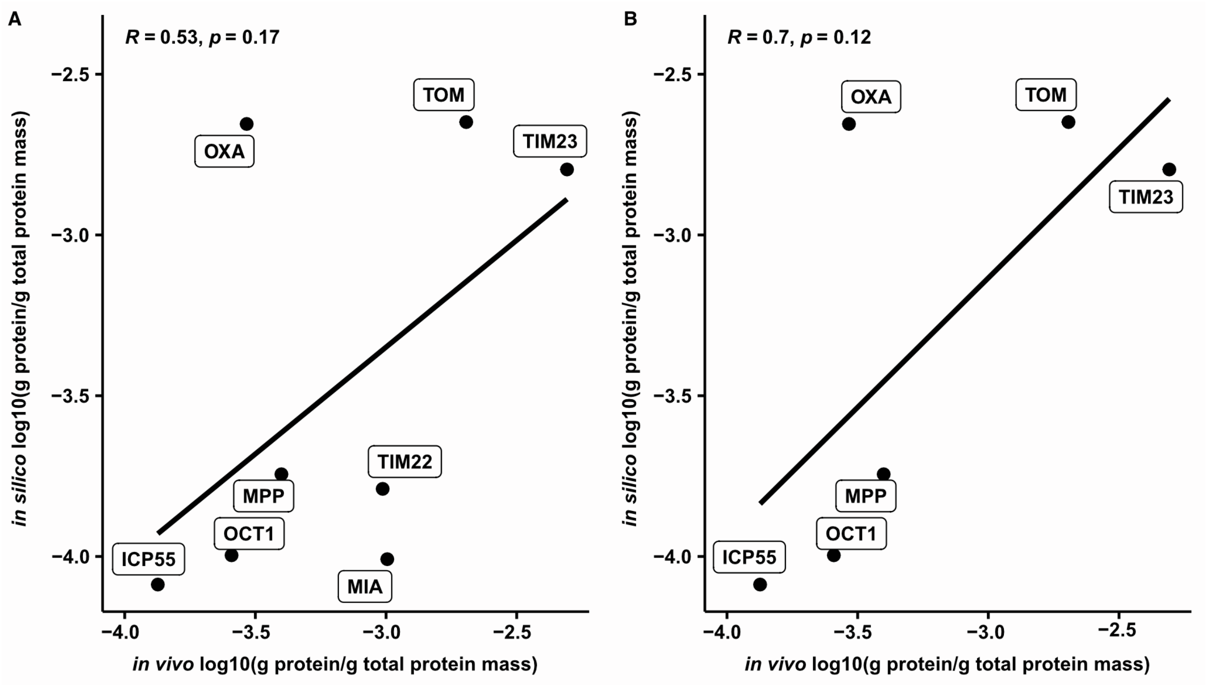

Figure S1. Comparison of *in silico* levels of import components with k_{cat} values estimated from proteomics data (Ho et al., 2018), related to Figure 3. (A) including all components and (B) excluding the main outliers MIA and TIM22. R, Pearson's correlation coefficient, p-values were calculated using a student's t-test.

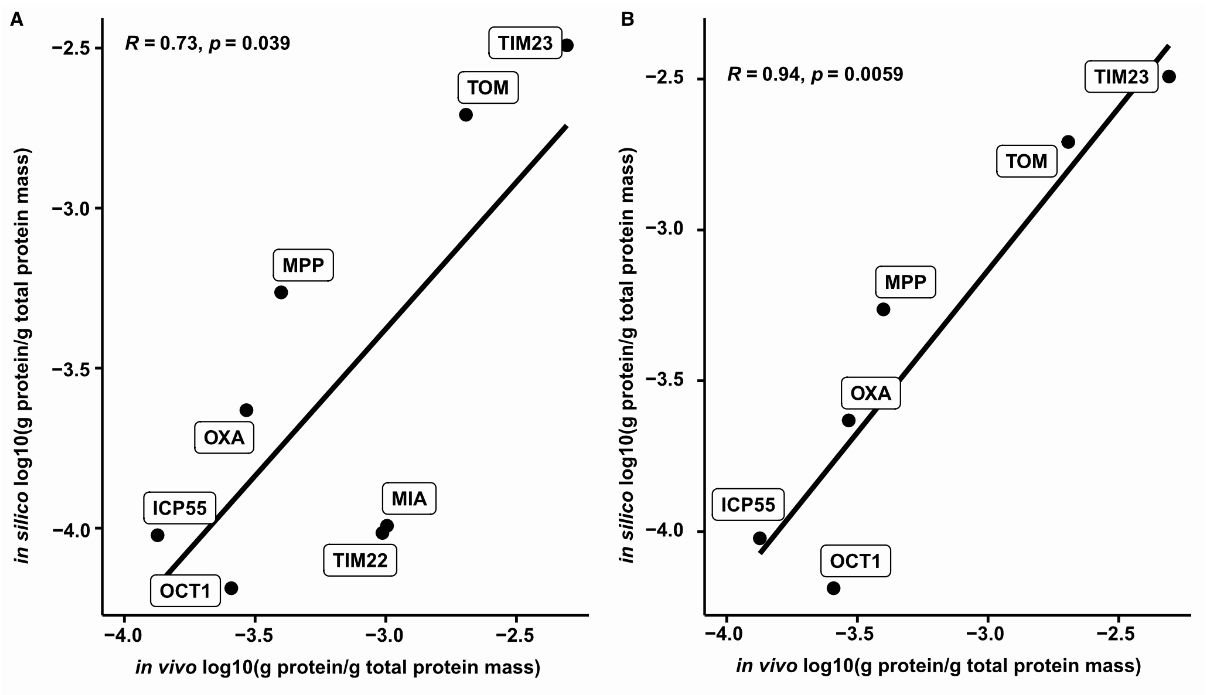

Figure S2. Comparison of *in silico* levels of import components with k_{cat} values curated using literature data, related to Figure 3. (A) including all components and (B) excluding the main outliers MIA and TIM22. R, Pearson's correlation coefficient, p-values were calculated using a student's t-test.

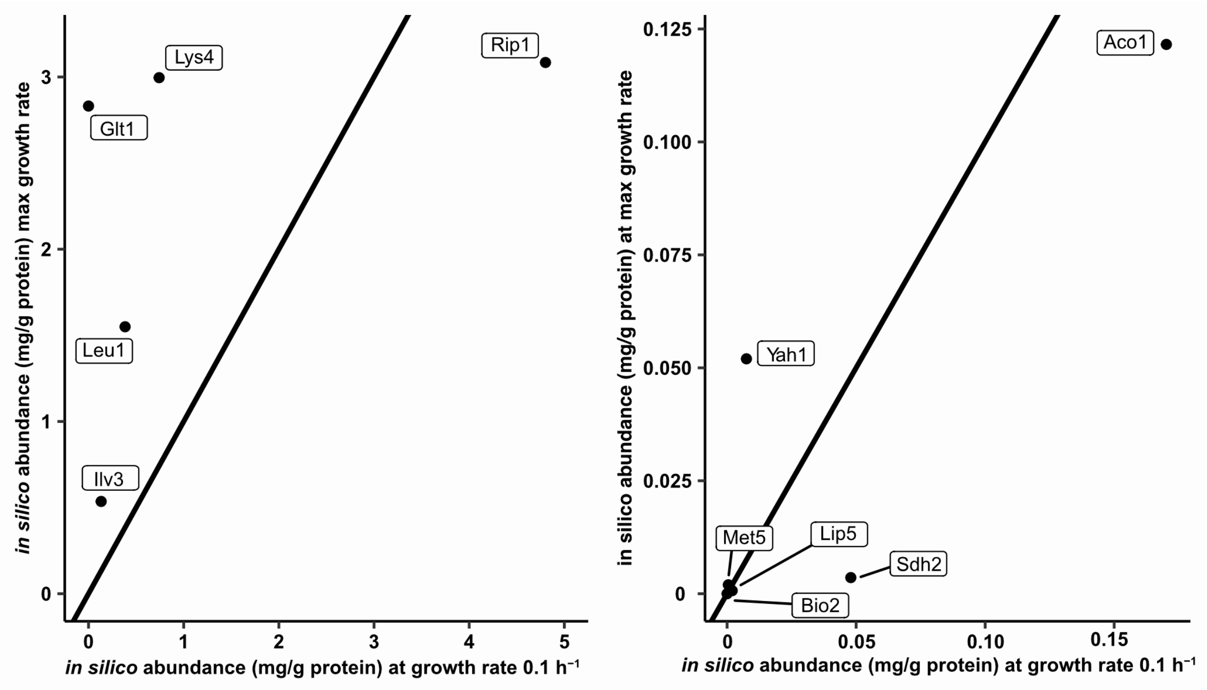

Figure S3. Comparison of *in silico* abundances of model proteins containing iron-sulfur clusters low (0.1 h^{-1}) and maximum growth rate, related to Figure 4.

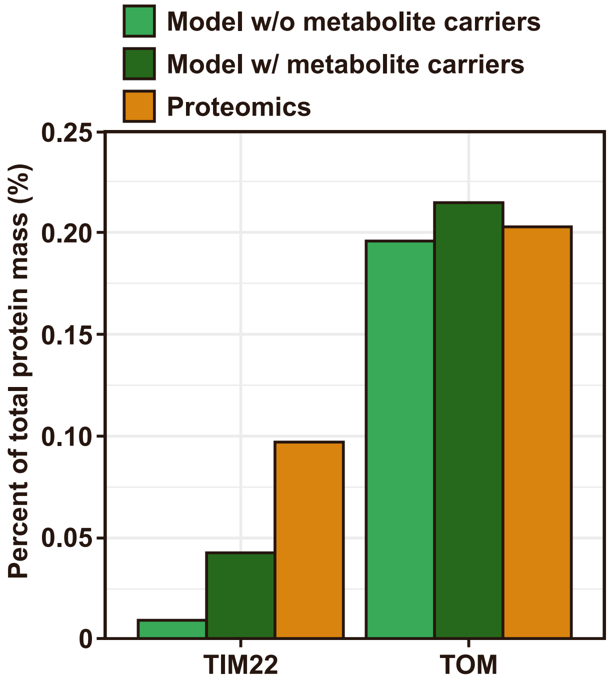

Figure S4. Comparison of model predictions before and after constraining metabolite carriers in model to proteomics data at D = 0.1 h^{-1}, related to Figure 3.
